# One-year clinical outcomes following theta burst stimulation for post-traumatic stress disorder

**DOI:** 10.1038/s41386-019-0584-4

**Published:** 2019-12-03

**Authors:** Nicholas J. Petrosino, Mascha van ’t Wout-Frank, Emily Aiken, Hannah R. Swearingen, Jennifer Barredo, Amin Zandvakili, Noah S. Philip

**Affiliations:** From the VA RR&D Center for Neurorestoration and Neurotechnology, Providence VA Medical Center, and The Department of Psychiatry and Human Behavior, Alpert Medical School of Brown University, Providence, RI 02903 USA

**Keywords:** Biomarkers, Outcomes research

## Abstract

Theta burst transcranial magnetic stimulation (TBS) is a potential new treatment for post-traumatic stress disorder (PTSD). We previously reported active intermittent TBS (iTBS) was associated with superior clinical outcomes for up to 1-month, in a sample of fifty veterans with PTSD, using a crossover design. In that study, participants randomized to the active group received a total of 4-weeks of active iTBS, or 2-weeks if randomized to sham. Results were superior with greater exposure to active iTBS, which raised the question of whether observed effects persisted over the longer-term. This study reviewed naturalistic outcomes up to 1-year from study endpoint, to test the hypothesis that greater exposure to active iTBS would be associated with superior outcomes. The primary outcome measure was clinical relapse, defined as any serious adverse event (e.g., suicide, psychiatric hospitalization, etc.,) or need for retreatment with repetitive transcranial magnetic stimulation (rTMS). Forty-six (92%) of the initial study’s intent-to-treat participants were included. Mean age was 51.0 ± 12.3 years and seven (15.2%) were female. The group originally randomized to active iTBS (4-weeks active iTBS) demonstrated superior outcomes at one year compared to those originally randomized to sham (2-weeks active iTBS); log-rank ChiSq = 5.871, df = 1, *p* = 0.015; OR = 3.50, 95% CI = 1.04–11.79. Mean days to relapse were 296.0 ± 22.1 in the 4-week group, and 182.0 ± 31.9 in the 2-week group. When used, rTMS retreatment was generally effective. Exploratory neuroimaging revealed default mode network connectivity was predictive of 1-year outcomes (corrected *p* < 0.05). In summary, greater accumulated exposure to active iTBS demonstrated clinically meaningful improvements in the year following stimulation, and default mode connectivity could be used to predict longer-term outcomes.

## Introduction

Post-traumatic stress disorder (PTSD) is a prevalent and impairing psychiatric disorder, marked by symptoms of intrusive re-experiencing, hyperarousal, avoidance behaviors, and altered mood and cognition [1, 2]. The impact of PTSD in military veterans is particularly high, and it is often comorbid with other psychiatric disorders, medical illnesses, disrupted social/occupational function, and increased suicidality [2–5]. Furthermore, current treatments are generally considered less effective in veterans [6], underscoring the need to develop novel interventions that can effectively reduce symptoms and maintain wellness over time.

Repetitive transcranial magnetic stimulation (rTMS) is an effective treatment for pharmacoresistant major depression [7, 8] and, when paired with symptom provocation, also efficacious for OCD [9]. While rTMS is safe and well-tolerated, standard treatment protocols may be burdensome to patients with a typical session lasting up to 37.5 min or longer [7]. Over the last several years there has been an increasing evidence base indicating a role of rTMS in the treatment of PTSD (reviewed in refs. [10, 11]). Studies using varied stimulation frequencies, sites of stimulation, and stimulation approaches (e.g., combination with symptom provocation or psychotherapy) have consistently indicated efficacy [12–16].

In an effort to further improve efficacy and tolerability, several novel forms of rTMS are being developed, and foremost among these is theta burst rTMS. This stimulation, which can be delivered in an intermittent (iTBS) or continuous fashion, involves a more rapid administration protocol with 50 Hz triplet bursts of stimulation repeated at 5 Hz in 2-s trains every 10 s [17]. When delivered as clinical treatment, the entire stimulation session requires 3–10 min (depending on the number of pulses delivered), thus demonstrating a significant advantage in terms of the administration time. Recent data has indicated noninferiority of iTBS for the treatment of pharmacoresistant major depression [18], as well as the potential use of theta burst to reduce craving in substance-use disorders [19].

In the context of this prior work, we recently reported the first study of iTBS for PTSD [20]. In this study, 50 veterans with PTSD initially received a resting state MRI scan, followed by two weeks of double-blind sham-controlled iTBS, followed by two weeks of unblinded (i.e., open-label) iTBS. Participants then returned 1-month later for further clinical assessments. Results included statistically significant superiority of active stimulation on social/occupational function at the end of the first two weeks, and, at 1-month found clinically meaningful and statistically significant reductions in PTSD, depression, and social & occupational function. In addition, we identified several patterns of pretreatment resting state functional connectivity associated with superior clinical outcomes; stronger within-network connectivity in the default mode network was predictive of later improvement, as was increased negative (anticorrelated) connectivity between the default mode and task-positive functional networks (e.g., executive, salience).

Given the chronic nature of PTSD and its relevant comorbidities, these results raised the important question as to whether the advantages of greater exposure to active iTBS persisted longer than the one-month endpoint of the parent study; such data would inform whether observations were clinically meaningful over the longer term. This is relevant since placebo effects were likely incorporated into shorter-term outcomes. Furthermore, since there are no multisite sham-controlled studies of iTBS for PTSD, results from longer-term observations could inform the design of future studies, particularly those incorporating longitudinal or maintenance approaches.

To this end, the present study examined naturalistic, 1-year clinical outcomes to test the hypothesis that greater cumulative exposure to active iTBS (i.e., 4-weeks vs. 2-weeks) would be associated with superior outcomes, using relapse as an operationally defined measure of clinical benefit. We also revisited baseline neuroimaging data from the parent study to explore whether pretreatment resting state functional connectivity could predict longer-term outcomes.

## Materials and methods

### Data collection

The Providence VA IRB approved all methods for this chart review. Electronic medical records were reviewed to evaluate naturalistic outcomes up to one year from the parent study’s endpoint; this endpoint was defined as a patient’s final day of participation. Included in the study were participants who completed at least the two-week double-blind iTBS phase. Please refer to Philip et al. (2019; NCT02769312) [19] for full inclusion criteria for the original cohort. Groups were defined by their original randomization (i.e., sham or active); those originally randomized to sham received two weeks of active stimulation (i.e., two weeks of sham were followed by two weeks of active iTBS) whereas those randomized to active stimulation received a total of four weeks of active iTBS unless otherwise indicated. Data abstraction was performed for each participant by reviewing relevant clinician notes. Primary outcome for this study was defined as clinical relapse, operationally defined as suicide (attempt or otherwise), inpatient psychiatric hospitalization, or need for rTMS retreatment. This definition was made to provide a measure of clinically relevant relapse. To determine suicide attempts or completions, notes were filtered by the text search “suicid[e]” and all occurrences were manually reviewed (NJP and EA, reviewed by AZV and NSP). For those patients who died in the study period, reason for death (chart, medical examiner notes, etc.) was reviewed for suicidality. To determine inpatient psychiatric hospitalizations, notes were filtered by the text searches “inpatient” and “admission” and all occurrences were manually reviewed as described above. Data on TMS retreatment were also collected from VA electronic records; relapse was defined as the date of patient-request or clinician-request to pursue rTMS retreatment.

Additional outcomes of interest included changes in medication use, efficacy of clinical TMS retreatment, and change in one-year no-show/cancellation rates. Changes in medication use were assessed using four categorical outcomes comparing one year to baseline: addition of new medication (antidepressants and second-generation antipsychotics), reduction of medication, switching of medication class, and change (addition, reduction, altered dosing) in as-needed benzodiazepine or antipsychotic prescriptions. Baseline medication data was collected from the parent study. Data on medications at one year were collected by reviewing active outpatient medications, as well as notes by treating psychiatrists throughout the following year. For those participants who received rTMS retreatment, the total number of sessions, as well as clinical outcomes measures (using the PTSD checklist for DSM-5 (PCL; [21]) and Inventory of Depressive Symptomatology-Self Report (IDSSR; [22]); used as standard measures in the rTMS clinic) at baseline and final rTMS session were reviewed.

### Data analysis

All data analyses were conducted on SPSS (v21, Armonk, NY). While demographic results were primarily taken from the parent study, an independent samples *t-*test and chi-square were performed for age and sex, respectively, for our subsample to evaluate any potential group differences prior to evaluating longer term outcomes. For primary outcome analyses, Kaplan-Meier survival curves were generated to compare time to clinical relapse between patients originally randomized to active vs. sham treatment, designated as 4-week and 2-week groups, respectively (with relapse as defined above); odds ratios comparing the two groups over the 1-year period were also generated. Chi-squared tests were conducted to assess for differences between groups in medication-related secondary outcomes of clinical interest, which included medication additions, medication reductions, medication class switches, and changes in as-needed benzodiazepine or antipsychotic prescriptions. No-show rates were calculated by manual review of the chart and compared to the year prior using *t*-tests. To determine clinical rTMS retreatment efficacy, the mean number of retreatment sessions and mean percent change in PCL-5 and IDSSR scores were calculated.

### MRI data collection, processing, and analyses

Baseline neuroimaging data were available for a subsample of participants (*N* = 26). Of these, thirteen participants were originally randomized to active and sham iTBS; ten participants relapsed in the 1-year post participation and 16 did not relapse. Full neuroimaging data collection and preprocessing procedures can be found in the Supplemental Information. In brief, data was acquired on a 3T Siemens MRI scanner, using a 32-channel head coil. A high-resolution T1-weighted structural image was collected from each participant (160 slices, TR = 1900 ms, TE = 2.98 ms, FOV = 2562 mm, and voxel size = 1.0 mm isotropic). Resting-state functional echo-planar imaging (EPI) data were collected following the structural scan (192 volumes, TR = 2500 ms, TE = 28 ms, FOV = 1922 mm, 42 slices, voxel size = 3.0 mm isotropic).

Data were preprocessed using the CONN Toolbox [23]. Structural data underwent tissue segmentation and normalization to Montreal Neurological Institute (MNI) Atlas space; fMRI preprocessing included: slice-time correction, realignment to the mean functional image, normalization to MNI Atlas space, and spatial smoothing. Per the recommendations of Ciric et al. [24], we implemented additional subject-level confound regression procedures to reduce the impact of head motion and non-neuronal signals on functional connectivity. Specifically, we identified volumes where excessive frame-to-frame motion (translation > 0.5, rotational > 0.005) or global signal variance (>3 SD) using the Artifact Detection Toolbox as implemented within CONN. These flagged volumes were included in a nuisance regression with six motion parameters, and their temporal derivates, estimated during realignment, and with five components each extracted from cerebrospinal fluid and white matter, following principal component deconstruction with anatomical CompCor [25, 26] within CONN. The resulting subject-level residuals were then bandpass filtered (0.008 > 0.1).

Exploratory seed-to-voxel analyses were performed using seeds derived from regions previously implicated in PTSD and iTBS, including the posterior cingulate cortex (representative of the default mode network), right dorsolateral prefrontal cortex (representing the executive control network and stimulation site), as well as the anterior insula and amygdala (representing the salience network) [27, 28]. The default mode, salience, and executive control networks and their functional neurocircuitry have been shown to be implicated in the pathology of PTSD [e.g., refs. 29–31]. Six millimeter seed spheres were constructed for 20 seeds in the default mode, executive control, and salience networks using the MarsBaR Toolbox (http://marsbar.sourceforge.net/). Central MNI coordinates for each seed were generated using the Neurosynth meta-analytic database [32]. Peak voxels were identified using construct terms associated with functional networks to generate whole-brain meta-analytic term maps (Executive: ‘working memory’; Default: ‘default’, Salience: ‘salience’). Because subgenual anterior cingulate cortex (sgACC) connectivity was not associated with clinical outcomes in the initial imaging results, it was not included as a seed in this exploratory analysis.

To identify predictive functional connectivity patterns, we contrasted whole brain resting-state functional connectivity seed maps between relapsers and non-relapsers at study baseline, after covarying a priori for participant age and scanner. We determined cluster significance using the voxel-height uncorrected threshold of *p* < 0.005 and cluster-level false discovery rate (FDR)-corrected threshold of *p* < 0.05. We then submitted significant clusters to an additional leave-one-out cross-validation procedure, following approaches used in prior work [33]. The resulting leave-one-out estimated parameters were then compared between relapsers vs. non-relapsers using a two-sample *t*-test. Only clusters where cross-validated t-statistics were significant at the two-tailed threshold of *p* < 0.05 are reported. To illustrate the direction of effect, we computed the average functional connectivity value across voxels for each cross-validated cluster and each group. *Z* scores were then converted back to Pearson’s *r* values for graphical representation.

## Results

Participant demographics are described in Table 1. Forty-six (92%) of the parent study’s 50 intent-to-treat participants were included in the present study. Of the 22 participants originally randomized to sham (2-week group), 21 received 10 active iTBS sessions, and one participant received nine active sessions (i.e., received during the unblinded phase of the study). Of the 24 participants originally randomized to active iTBS (4-week group), 22 received 20 active iTBS sessions, and one participant received 17 and another received 19. The mean age was 51.0 ± 12.3 years and seven (15.2%) were female. The groups were balanced in terms of sample size, age (*p* = 0.16), and sex (*p* = 0.78).

**Table 1:** Demographics and primary/secondary outcomes by group.

	Overall (*N* = 46)	Sham iTBS (*n* = 22)	Active iTBS (*n* = 24)
Demographics			
Age, Mean (SD)	51.0 (12.3)	53.6 (11.0)	48.5 (13.1)
Females, *N* (%)	7 (15.2%)	3 (13.6%)	4 (16.7%)
Primary outcome			
Clinical relapse, *N* (%)	22 (47.8%)	14 (63.6%)	8 (33.3%)
TMS retreatment	18 (39.1%)	11 (50.0%)	7 (29.2%)
Psychiatric hospitalizations	3 (6.5%)	2 (9.1%)	1 (4.2%)
Suicide attempts	0	0	0
Death	1 (2.22%)^a^	1 (4.5%)^a^	0
Days to clinical relapse, Mean (SE)	241.4 (20.9)	182.0 (31.9)	296.0 (22.1)
Secondary outcomes			
Medication changes, *N* (%)			
Medication additions	12 (26.1%)	5 (22.7%)	7 (29.2%)
Medication reductions	11 (23.9%)	4 (18.1%)	7 (29.2%)
Medication class switch	2 (4.3%)	1 (4.5%)	1 (4.2%)
Change PRN benzo/antipsych	5 (10.9%)	2 (9.1%)	3 (12.5%)
Clinical TMS retreatment, *N* (%)	7 (15.2%)	3 (13.6%)	4 (16.7%)
Δ No-shows/cancel, Mean (SD)	0.52 (5.42)	1.41 (5.53)	−0.29 (5.31)

**Fig. 1:**
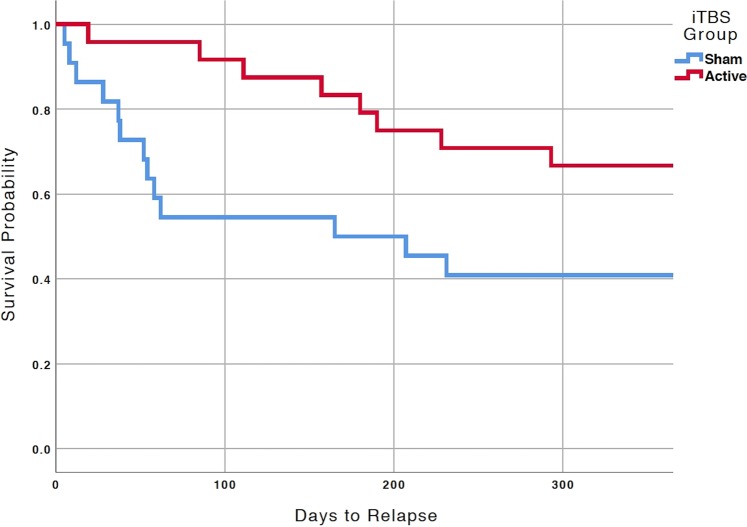
Survival curve analysis comparing groups at one year. Kaplan-Meier survival curves demonstrating superiority of active iTBS over sham stimulation in the year post-participation. Log-rank ChiSq = 5.871, df = 1, *p* = 0.015; OR = 3.50, 95% confidence interval 1.04–11.79. In the original study [20], participants randomized to the active group received 4-weeks of active iTBS, whereas those randomized to sham received 2-weeks of active stimulation. Abbreviations: iTBS, intermittent theta burst stimulation.

Groups are described further based on their original randomization; those randomized to sham received two weeks of active stimulation (i.e., two weeks of sham followed by two weeks of active iTBS), whereas those randomized to active stimulation received four weeks of active iTBS. Please see Fig. 1 for statistical reporting related to all-cause relapse rates (significant at *p* < 0.015); there were no significant group differences in secondary outcomes.

*iTBS* intermittent theta burst stimulation, *SD* standard deviation, *SE* standard error, *PRN* as needed, *benzo* benzodiazepines, *antipsych* antipsychotics

^a^Death from overdose

Overall, 22 (47.8%) patients demonstrated clinical relapse by one year: one death by overdose (fentanyl/alcohol), three (6.5%) inpatient psychiatric hospitalizations, and 18 (39.1%) TMS retreatments. Fourteen (63.6%) experienced clinical relapse in the 2-week group vs. eight (33.3%) in the 4-week group. Kaplan-Meier survival curves (Fig. 1) demonstrated superior outcomes at one year in the 4-week group (log-rank ChiSq = 5.871, df = 1, *p* = 0.015). Mean number of days to clinical relapse was 182.0 ± 31.9 in the 2-week group vs. 296.0 ± 22.1 in the 4-week group. The odds of clinical relapse were significantly greater in the 2-week group (OR = 3.50, 95% confidence interval 1.04–11.79).

Chi-square analyses of medication additions, medication reductions, medication class switches, and changes in PRN benzodiazepines or antipsychotics showed no significant difference between groups, and there were no significant changes in the rates of no-shows to appointments compared to the prior year (all *p* > 0.1). Eighteen patients (39.1% of the total followed for 1-year) requested neuromodulation retreatment in the subsequent year; of those eleven were in the 2-week active iTBS group and seven were in the 4-week active iTBS group. PTSD and depressive symptom severity, as well as time to retreatment request, are described in Table 2. Of these, *n* = 7 patients eventually received clinical rTMS; in retreated patients the mean number sessions was 18.6 ± 10.0 and clinical scores overall decreased with retreatment (48.6 ± 97.7% for the PCL-5, 31.4 ± 64.4% for the IDSSR). The remainder received other treatment or research participation. Statistical testing comparing change in clinical scores across these subsequent time points was not performed due to insufficient power.

**Table 2:** Clinical variables for the *n* = 18 participants that requested rTMS retreatment.

iTBS group^a^	Baseline^b^	End of study participation		Follow-up-retreatment request	
		2-week (*n* = 11)	4-week (*n* = 7)	2-week (*n* = 11)^c^	4-week (*n* = 7)^c^
PTSD symptom severity					
PCL-5, mean total score (SD)	49.68 (10.31)	40.50 (18.83)	29.14 (12.40)	46.83 (13.66)	40.43 (15.91)
Depressive symptomatology					
IDSSR, mean total score (SD)	41.02 (11.74)	33.67 (15.61)	34.29 (20.42)	34.25 (15.90)	52.67 (18.50)
QIDS, mean total score (SD)		–	–	15.75 (2.82)	10.75 (5.25)
Elapsed time					
Mean days (SD)		–	–	88.75 (87.07)	126.14 (78.32)

*iTBS* intermittent theta burst stimulation, *SD* standard deviation, *PTSD* post-traumatic stress disorder, *PCL-5*, PTSD checklist for DSM-5, *IDSSR* inventory of depressive symptomatology, self-report, *QIDS* quick inventory of depressive symptoms (self-report)

^a^Groups (2-week and 4-week) indicate the number of weeks of active iTBS

^b^Baseline variables are presented as group means; Baseline PCL-5 mean (SD) in the 2-week and 4-week groups were 50.0 (11.4) and 49.4 (9.4), respectively.; Baseline IDSSR scores in the 2-week and 4-week groups were 39.2 (11.50) and 42.8 (11.90), respectively. No significant baseline differences (all *p* > 0.05) were observed

^c^Depression rating scale data available from IDSSR (*n* = 4, 3) and QIDS (*n* = 8,4) for subjects that completed either 2-weeks or 4-weeks of iTBS, respectively. Whereas at last TBS evaluation, all subjects completed the IDSSR (*n* = 12 and 7, for 2-weeks or 4-weeks, respectively)

**Fig. 2:**
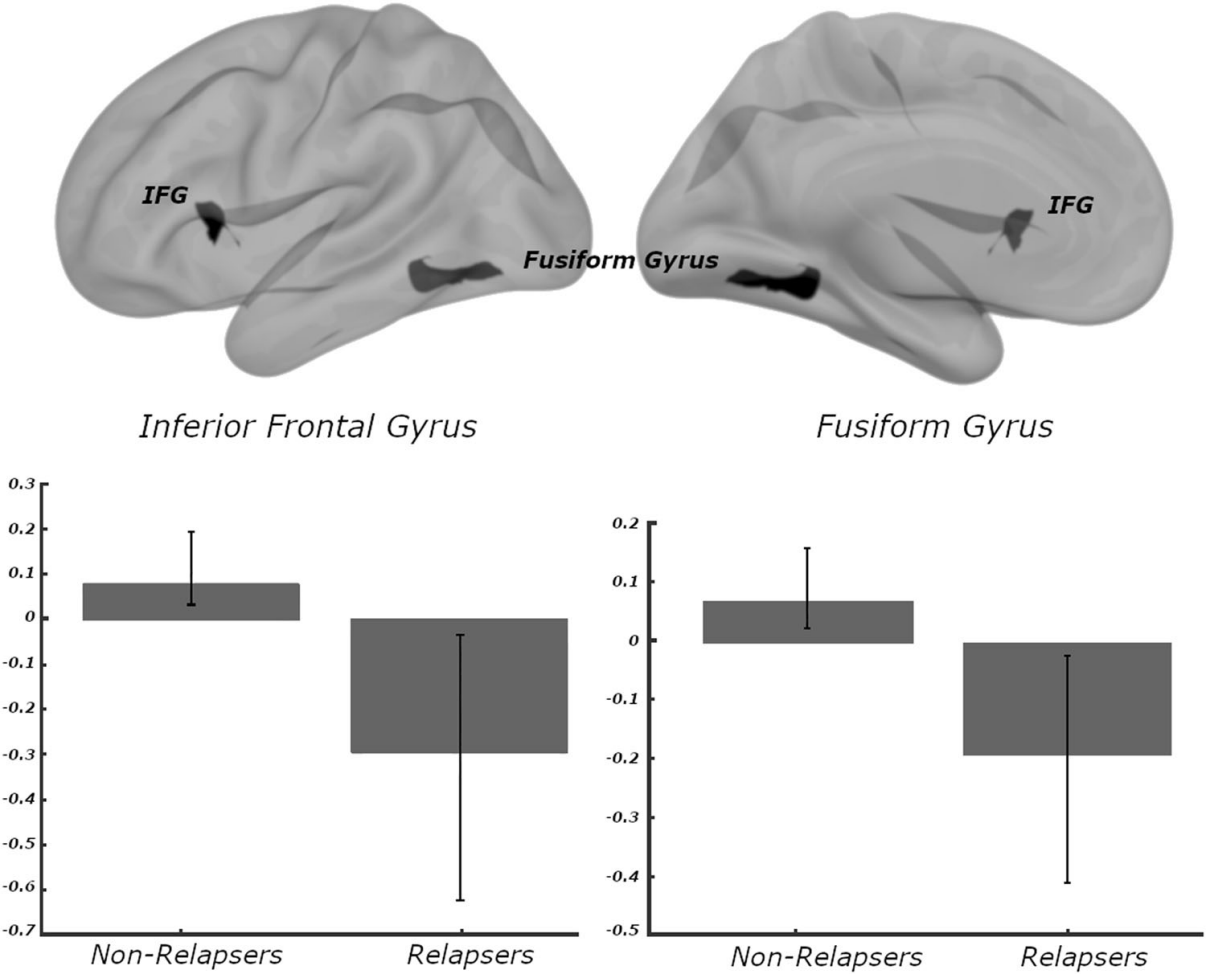
Posterior cingulate cortex connectivity predicts 1-year outcomes. Baseline resting-state functional connectivity relationships associated with 1-year outcomes. For ease of interpretation, all Fisher's *Z*-scores have been converted back into Pearson's *r* values. Posterior cingulate cortex and IFG (pars opercularis) were positively correlated in non-relapsers (mean *r* = 0.09, SEM = 0.04), but anti-correlated in participants that eventually relapsed (mean *r* = −0.31, SEM = 0.03). Similarly, we observed positive connectivity between posterior cingulate and fusiform gyrus cluster in non-relapsers (mean *r* = 0.07, SEM = 0.02), but negative connectivity in relapsers (mean *r* = −0.19, SEM = 0.02). IFG inferior frontal gyrus.

Neuroimaging analysis revealed that functional connectivity of the posterior cingulate cortex (PCC) was predictive of one-year outcomes. These included two regions that differed between participants that did, or did not, relapse at year post-treatment. These clusters were located in the inferior frontal gyrus (pars opercularis; MNI: −48, 14, 08; *p*-FDR < 0.05; cross-validated at *p* = 0.02), and fusiform gyrus (MNI: −28, −56, −14; *p*-FDR < 0.05; cross-validated at *p* < 0.001). Of note, the direction of effects was generally consistent; participants who relapsed had greater negative (i.e., anticorrelated) PCC resting state functional connectivity (Fig. 2). When considering only those that received active stimulation, similar findings were observed in that more negative connectivity was consistently associated with relapse.

## Discussion

Our naturalistic one-year follow-up of veterans with PTSD who received iTBS demonstrated that greater cumulative iTBS exposure is associated with better longer-term outcomes. Both the Kaplan-Meier survival analysis and odds ratios of relapse demonstrate clinically meaningful superiority of four weeks of active iTBS (i.e., those originally randomized to active stimulation). Furthermore, not only were participants in the 2-week group (i.e., those originally randomized to sham) more likely to relapse in one year, they also did so sooner. This likely reflects a dose-dependent durability of response of iTBS, beyond the placebo effects that were likely incorporated into shorter-term outcomes. Furthermore, those retreated with standard rTMS demonstrated significant reductions in PTSD and depressive symptoms, which is consistent with high rates of retreatment success in rTMS for major depression [34, 35].

These findings are consistent with our previous study [20] that showed that, while the majority of clinical benefits of iTBS occurred in the first week that a participant received active stimulation, exposure to additional stimulation did not appear to provide further reduction in clinical symptoms. Yet at 1-month, participants in the 4-week group had superior clinical outcomes. In other words, while additional iTBS exposure did not increase the maximal efficacy, it may increase the durability of achieved improvements. The present study provides support for this finding and shows that the durability may extend well beyond one month after treatment; indeed, up to another subsequent year. Yet, these differences did not extend to other clinical variables of interest, such as medication use or broader treatment participation, although to what degree these variables are influenced by nonspecific elements in clinical care remains beyond the scope of the current report. Since there are no sham-controlled multisite studies of iTBS for PTSD, this data may provide insights into the design of future clinical trials.

The TMS retreatment rate observed in the 4-week group (33.3% in one year) is comparable to those observed in similar studies of standard rTMS for depression. For example, a multisite, naturalistic, one-year follow-up study on the durability of response of 10 Hz TMS in 257 patients with treatment-resistant major depression found that 36.2% required retreatment with rTMS [34]. These outcomes are superior to long-term efficacy when using rTMS as a monotherapy (i.e., in medication-free, treatment-resistant individuals), of which two thirds may require retreatment [35]. This would be expected given the vast majority of participants in the parent study were receiving concurrent medications. These data also suggest promise for future studies to prospectively evaluate the efficacy of retreatment or maintenance iTBS for PTSD.

The exploratory neuroimaging results indicated several important findings. The fusiform gyrus and ventrolateral prefrontal cortex play key roles in contextual processing [36, 37] and strategic memory operations [38], respectively, cognitive operations used for safety learning and are relevant in PTSD. While interpretations about the directionality of these findings remain speculative, our results suggest that resting connectivity between default network subregions is associated with superior longer-term outcomes. This finding is consistent with previously reported imaging correlates of 1-month outcomes [20]. Though we cannot completely discount the influence of nonspecific group effects at one month, the temporal consistency of this pattern over one year following stimulation indicates that default network connectivity may exert considerable influence over longer-term clinical outcomes with iTBS.

Like most other neuroimaging research of rTMS, and regardless of the diagnoses treated, default mode network connectivity was implicated in treatment response [39, 40, reviewed in ref. 41], underscoring the near-term potential to use this connectivity profile in clinical decision-making; whether these markers are robust enough to inform decisions about whether to utilize iTBS or another intervention for PTSD remains an important and unanswered question. Future studies of whether these imaging profiles change with treatment and eventual relapse are needed to better characterize whether network connectivity can be used a reliable marker of longer-term treatment response, or perhaps a prognostic indicator that can be used to identify patients in need of retreatment of maintenance stimulation.

This study has several limitations; even though the parent study is the largest study of iTBS for PTSD, the sample size remains modest and the participants were demographically homogenous. Importantly, the 1-year follow up period was not controlled (i.e., veterans returned to general clinical care), so these results can only be interpreted as naturalistic. We also were not able to reliably disentangle the reason for relapse (i.e., related to PTSD or comorbidities in the patient sample including depression and substance use, etc.) Data was exclusively collected from the VA’s electronic health record, thus it is possible clinical relapse events and other relevant data occurring at outside hospitals may have been missed, although extensive manual review of patient records should have provided some indication of non-VA care. Symptom severity was also not systematically measured during clinical care in all patients over the year after participation in the original iTBS trial. To mitigate this issue, we utilized “clinical relapse” as a primary outcome incorporating clinically meaningful events that could be reliably detected on chart review. Furthermore, this study was underpowered to evaluate imaging interactions between relapse status and stimulation group, and evaluating the stability of biomarkers of stimulation response is an important area of further inquiry.

In summary, our naturalistic follow-up study demonstrated that greater cumulative exposure to active iTBS was associated with superior outcomes the year after treatment, and our exploratory imaging findings support default mode network connectivity as a potential predictor of longer-term TBS outcomes. Observed relapse rates were consistent with the broader literature on TMS for pharmacoresistant major depression, and retreatment rates were also consistent with this literature. Further investigation, ideally with prospective data and in non-VA patients, is warranted to confirm our findings. Those caveats aside, this data provides additional real-world support of the use of iTBS for PTSD in this difficult-to-treat patient population.

## Funding and disclosure

Effort on this paper was supported in part by the VA RR&D Center for Neurorestoration and Neurotechnology, Department of Veterans Affairs grants I01 RX002450 (NSP, MvWF) and IK2 CX001824 (JB); NIH grants U54 GM115677 (AZ), and P20 GM130452 (NSP, MvWF). The funders had no role in the conduct of the study, paper preparation, or the decision to submit for publication. The views expressed in this article are those of the authors and do not necessarily reflect the position or policy of the funders. The authors report no biomedical conflicts of interest related to this work. In the past three years, Dr. Philip has received grant support from Neuronetics and Neosync through clinical trial contracts and has been an unpaid scientific advisory board member for Neuronetics. The remaining authors declare no conflict of interest.

## Supplementary information

Supplemental material
