# Supplementary material for: One-year clinical outcomes following theta burst stimulation for post-traumatic stress disorder
Source: Neuropsychopharmacology. 2019 Dec 3;45(6):940–6. doi: 10.1038/s41386-019-0584-4 (PMC7162862; doi:10.1038/s41386-019-0584-4)
Supplement: Supplementary file 1 — Supplemental material [file 41386_2019_584_MOESM1_ESM.docx]

SUPPLEMENTAL INFORMATION

MRI Data Collection and Preprocessing

MRI data were collected at either the Brown University MRI Research Facility on a Siemens (Erlangen, Germany) 3T Prisma MRI scanner (n=15) or at the Providence VA Medical Center on the Siemens 3T Verio MRI Scanner (n=11) using a 32-channel head coil. A high-resolution T1-weighted structural image was collected from each participant (160 slices, TR=1,900ms, TE=2.98ms, FOV=2562 mm, and voxel size = 1.0 mm isotropic). “Resting-state” functional echo-planar imaging (EPI) data were collected following the structural scan (192 volumes, TR=2,500ms, TE=28ms, FOV= 1922 mm, 42 slices, voxel size=3.0mm isotropic). During resting-state functional imaging, participants were instructed to stay as still as possible while visually fixating on a white crosshair presented on a black foreground.

MRI data were preprocessed using the CONN Toolbox for functional MRI (Whitefield-Gabrielli et al., 2012). Structural data underwent tissue segmentation and normalization to Montreal Neurological Institute (MNI) Atlas space; fMRI preprocessing included: slice-time correction, realignment to the mean functional image, normalization to MNI Atlas space, and spatial smoothing. We applied several additional preprocessing steps to subjects’ fMRI data to reduce the impact of motion artifacts and non-neuronal signals on estimates of functional connectivity. We first identified high-motion (translation>0.5, rotational>0.005) and high global signal variance (>3SD) volumes with the Artifact Detection Toolbox included in CONN. These flagged volumes were then included in a nuisance regression with six motion parameters estimated during realignment and their first temporal derivatives, and with five components each extracted from cerebrospinal fluid and white matter per anatomical CompCor. Subject-level was then bandpass filtered (0.008>0.1) after nuisance regression.

Seed-to-Voxel Procedures

To identify predictive functional connectivity patterns, we contrasted whole brain resting-state functional connectivity seed maps between relapsers and non-relapsers at study baseline after covariance for age and scanner. We determined cluster significance using the voxel-height uncorrected threshold of p<.005 and cluster-level false discovery rate (FDR)-corrected threshold of p<.05. We submitted significant clusters to an additional leave-one-out cross-validation procedure given our use of a more liberal voxel-level threshold (following methods in Philip et al., 2018). The resulting leave-one-out estimated parameters were then compared between relapsers vs. non-relapsers using a two-sample t-test. Only clusters where cross-validated t-statistics were significant at the two-tailed threshold of p<.05 are reported. To illustrate the direction of effect, we computed the average functional connectivity value across voxels for each cross-validated cluster and each group. For ease of interpretation, Z scores were converted back to Pearson’s r values which are plotted in Figure 2 along with means and standard errors.

REFERENCES

1. Whitfield-Gabrieli S, Nieto-Castanon A. Conn: a functional connectivity toolbox for correlated and anticorrelated brain networks. *Brain Connect*. 2012;2(3):125-141.
2. Philip NS, Barredo J, van ‘t Wout-Frank M, Tyrka AR, Price LH, Carpenter LL. Network Mechanisms of Clinical Response to Transcranial Magnetic Stimulation in Posttraumatic Stress Disorder and Major Depressive Disorder. *Biol Psychiatry*. 2018;83(3):263-272.
